# Perioperative blood loss in cardiac surgery: Validation of machine learning-derived clusters

**DOI:** 10.1177/02676591261440347

**Published:** 2026-03-26

**Authors:** Yerlan Orazymbetov, Serik Aitaliyev, Povilas Jakuška, Audronė Veikutienė, Tadas Lenkutis, Rassul Zhumagaliyev, Aušra Saudargienė, Rimantas Benetis

**Affiliations:** 1 Department of Cardiac, Thoracic and Vascular Surgery, Hospital of Lithuanian University of Health Sciences Kauno Klinikos, 230647 Lithuanian University of Health Sciences, Kaunas, Lithuania; 2 487619 National Scientific Medical Centre, Astana, Kazakhstan; 3 Institute of Cardiology, 230647 Lithuanian University of Health Sciences, Kaunas, Lithuania; 4 Department of Anaesthesiology, Medical Academy, 230647 Lithuanian University of Health Sciences, Kaunas, Lithuania; 5 Neuroscience Institute, 230647 Lithuanian University of Health Sciences, Kaunas, Lithuania

**Keywords:** blood loss, cardiac surgery, chest tube output, Hb/kg index, machine learning-derived clusters

## Abstract

**Background:**

There is no universally accepted definition of perioperative blood loss in cardiac surgery. Existing methods are based on chest tube output and are not normalised for patient weight.

**Objective:**

To validate machine learning-derived blood loss severity clusters based on a haemoglobin mass loss per kilogram (Hb/kg index).

**Methods:**

This single-center prospective study included 195 patients undergoing cardiac surgery between October 2023 and November 2024. Three clusters derived using K-Medoids were mapped to the Hb/kg index to define cut-offs. Cluster discrimination was assessed by receiver operating characteristics (ROC) analysis (area under the curve (AUC)). Group comparisons were performed using analysis of covariance adjusted for age and gender. Associations between the Hb/kg index and clinical outcomes, including transfusion requirements and complications were analysed using Chi-square tests and adjusted two-way Analysis of Covariance (ANCOVA).

**Results:**

Clustering identified three groups (Mild, Moderate, Severe) defined by optimal Hb/kg thresholds of 1.72 and 2.10. The Severe cluster demonstrated strong discriminative performance (AUC = 0.790, 95% confidence interval 0.721–0.859). Chest tube output did not differ significantly between clusters (*p* = 0.097), while haemoglobin mass loss through chest tubes demonstrated a significant effect (*p* = 0.011).

**Conclusions:**

The Hb/kg Index is a validated, data-driven, objective metric for perioperative blood loss, offering greater precision than traditional chest tube drainage volume. It effectively stratifies bleeding severity and identifies high-risk patients with lower BMI.

## Introduction

Bleeding in cardiac surgery remains a significant clinical burden with a reported incidence ranging from 8% to 15%.^1–3^ Excessive bleeding is associated with an eight-fold increase in the risk of death.^4^ Its impact on patient outcomes is second only to low cardiac output syndrome, perioperative stroke, and renal failure.^5,6^ Furthermore, bleeding leads to increased rates of reoperation, higher transfusion requirements and longer hospital stays.^7,8^ Despite this clinical significance, there is no universally accepted definition of blood loss. Several classification systems have been developed based on chest tube volume, blood transfusions, or reoperations.

For instance, the Kirklin and Barratt-Boyes criteria recommend immediate surgical re-exploration if the chest tube drainage (CTD) exceeds 5 mL/kg in any single hour or 3 mL/kg/hour for three consecutive hours.^9^ Some authors define active bleeding as blood loss exceeding 1.5 mL/kg/hour for six consecutive hours,^10^ while others suggest blood loss of more than 200 mL within 1 hour.^11^ In contrast, other clinical protocols, including those at Massachusetts General Hospital, use weight-adjusted volume guidelines (mL/kg) to trigger intervention.^12^ Although these tools have prognostic value, they share significant limitations.^13–15^ Visual estimation of chest tube drainage (CTD) is inaccurate; the fluid in the CTD contains a mixture of blood and serum, which can be under- or overestimated.^10,11,16,17^ Moreover, conventional estimation methods ignore patient weight. For example, the same 1000-mL blood loss has markedly different haemodynamic implications for a 60 kg patient compared to 100 kg. To address this gap, we previously introduced the Hb/kg Index, a physiological metric that quantifies haemoglobin mass loss normalised for patient weight.^18^ While this method provides an accurate calculation of blood loss, its clinical discriminatory ability remains unvalidated. Therefore, this study aimed to validate the Hb/kg Index using machine learning-derived clusters. We hypothesised that cluster analysis would identify objective bleeding severity groups (Mild, Moderate, Severe) associated with distinct physiological profiles and clinical requirements.

## Materials and methods

### Study design and population

This prospective observational study was conducted in the Department of Cardiac Surgery and the Department of Anaesthesiology (Intensive Care Unit) at the Hospital of the Lithuanian University of Health Sciences, Kaunas, Lithuania, from October 2023 to November 2024. A total of 195 adult patients undergoing elective cardiac surgery with cardiopulmonary bypass (CPB) were included. Exclusion criteria were emergency or urgent surgery, re-sternotomy for bleeding or cardiac tamponade, aortic dissection, heart transplantation, implantation of a mechanical circulatory support system, haematological disorders, and hepatic or renal failure.

### Ethical approval

The study was approved by the Kaunas Regional Biomedical Research Committee (Approval No. BE-2-53, August 2023). Written informed consent was obtained from all participants prior to inclusion.

### Data collection

All procedures were performed under general anaesthesia and CPB according to standard institutional protocols. Demographic and anthropometric data (age, gender, weight, BMI) were recorded. Serial laboratory assessments, including complete blood count (Hb, Hct, RBC, platelets) and coagulation profiles (PT, aPTT, INR, fibrinogen, D-dimer), were performed at four time points: baseline (T0), immediately postoperatively (T1), and at 6 (T6) and 18 (T18) hours. CTD volume was monitored for 18 h following ICU admission, with simultaneous fluid sampling for Hb mass analysis. Antiplatelet and anticoagulant drugs were discontinued 5–7 days before surgery. The follow-up period continued until hospital discharge. Blood samples were analysed using an automated haematology analyser (Advia 2120i, Siemens Healthcare).

### Study outcomes

The primary outcome was the development and validation of data-driven thresholds for the Hb/kg Index using K-Medoids clustering to provide a weight-normalised alternative to volume-based metrics. A key component of this validation was the comparative analysis between total CTD volume and the actual haemoglobin mass loss contained within that fluid. Secondary outcomes included clinical and physiological indicators, including transfusion requirements and the incidence of postoperative complications in terms of heart failure (HF), atrial fibrillation (AF), and acute kidney injury (AKI).

### Statistical analysis

Data were analysed using IBM SPSS Statistics (version 30.0) and Python 3.10. Continuous variables are presented as mean ± SD, and categorical variables as frequencies (%). Patients were classified into three severity groups using K-Medoids clustering based on the Hb/kg index. To ensure clinical relevance, variables for clustering were selected based on significant Spearman’s correlation with the Hb/kg index. Data-driven cut-off thresholds were calculated as midpoints between the median values of adjacent clusters, defining distinct Mild, Moderate, and Severe blood loss categories. Discriminative performance was assessed using Receiver Operating Characteristic (ROC) analysis and the Area Under the Curve (AUC). Differences between clusters were evaluated using two-way Analysis of Covariance (ANCOVA), adjusted for age and gender to control for confounding, and Chi-square tests for categorical variables. Statistical significance was defined as p < 0.05. Detailed methodology regarding normality testing, outlier management (3 × IQR rule), feature selection, and effect size calculations is provided in the Supplemental Material.

## Results

### Study cohort and cluster identification

The cohort included 195 patients (69% men). K-Medoids analysis, using 28 variables significantly correlated with the Hb/kg Index (Supplemental Table 1), identified three distinct blood loss severity clusters (Table 1). Based on data-driven thresholds (1.72 and 2.10 Hb/kg), patients were stratified into Mild (Hb/kg ≤ 1.72), Moderate (1.72 < Hb/kg ≤ 2.10), and Severe (Hb/kg > 2.10) blood loss groups. Baseline characteristics are presented in Table 2. Patients in the Severe cluster had significantly lower BMI (*p* = 0.015). Importantly, after controlling for age and gender using adjusted two-way ANCOVA, this association became highly significant (*p* < 0.001, Table 3). There were no statistically significant differences regarding the type of surgery (*p* = 0.051). Analysis of longitudinal haemoglobin dynamics showed that the Severe cluster had a baseline haemoglobin of 144.5 ± 11.7 g/L, which declined by 46 g/L to 98.4 g/L at 18 h. The Mild cluster had significantly lower baseline levels (134.5 ± 14.6 g/L; *p* < 0.001, Table 2) and dropped by 27 g/L to 107.5 g/L at 18 h. The overall prevalence of allogeneic red blood cell transfusion was 7.2% (14/195 patients).

**Table 1.** Descriptive Statistics of Patient Clusters based on the Hb/Kg Index.

Clusters	*n*	Severity level of blood loss	Hb/kg range	Median	Mean
Cluster 1	76	Mild	≤1.72	1.65	1.71
Cluster 2	46	Moderate	1.72 < Hb/Kg ≤ 2.10	1.80	1.76
Cluster 3	73	Severe	>2.10	2.41	2.44

*Note*: Hb/kg – haemoglobin mass loss per kilogram of patient weight.

**Table 2.** Patient characteristics (*n* = 195).

Characteristics	Mild group (*n* = 76)	Moderate group (*n* = 46)	Severe group (*n* = 73)	*p*-value
Age (years), mean ± SD	65.2 ± 7.2	66.4 ± 6.8	66.1 ± 7.2	0.665
Sex ratio, male/female, n	44/32	36/10	54/19	0.030
Weight (kg), mean ± SD	89.4 ± 18.2	84.7 ± 13.6	78.9 ± 14.5	0.024
BMI (kg/m2), mean ± SD	30.5 ± 5.3	28.7 ± 4.9	26.3 ± 4.1	0.015
EuroSCORE II, mean ± SD	2.98 ± 2.78	3.07 ± 3.38	2.61 ± 2.72	0.815
LVEF, mean ± SD	51.5 ± 6.3	50.9 ± 8.2	49.4 ± 8.1	0.496
Type of surgery				0.051
CABG, n (%)	31 (40.8%)	28 (60.9%)	42 (57.5%)	
Valve replacement/repair, n (%)	31 (40.8%)	8 (17.4%)	18 (24.7%)	
Combined surgery (CABG + valve), n (%)	14 (18.4%)	10 (21.7%)	13 (17.8%)	
CPB Duration (min), mean ± SD	98 ± 35	97 ± 32	96 ± 33	0.925
Laboratory parameters				
Hb concentration at T0, (g/L)	134.5 ± 14.6	140.2 ± 10.3	144.5 ± 11.8	<0.001
Hb concentration at T1, (g/L)	115.3 ± 14.0	111.0 ± 10.7	106.1 ± 12.5	<0.001
Hb concentration at T18, (g/L)	107.5 ± 12.7	104.9 ± 16.0	98.4 ± 14.6	<0.001

*Note*: BMI – Body Mass Index; CABG – Coronary Artery Bypass Grafting; CPB – Cardiopulmonary Bypass; Hb – Haemoglobin, LVEF – Left Ventricular Ejection Fraction; SD – Standard Deviation; EuroSCORE – European System for Cardiac Operative Risk Evaluation; Time periods: T0 – before surgery; T1 – immediately after surgery; T18 – 18 h after surgery.

**Table 3.** Comparison of Continuous variables across blood loss severity clusters (ANCOVA).

No.	Variables	F	df1	df2	*p*-value	$\eta_{p}^{2}$	R^2^	Adj.R^2^
1	Hb/kg	250.435	2	186	0.000	0.729	0.803	0.797
2	Weight	9.811	2	188	0.0001	0.095	0.224	0.199
3	BMI	10.322	2	188	0.0001	0.099	0.148	0.120
4	Hb T0	8.128	2	188	0.0004	0.080	0.223	0.198
5	Hb T1	7.863	2	188	0.0005	0.077	0.325	0.304
6	Hb T18	5.691	2	187	0.0040	0.057	0.283	0.260
7	RBC T1	11.853	2	188	0.000	0.112	0.273	0.249
8	RBC T6	4.087	2	188	0.0183	0.042	0.162	0.135
9	RBC T18	6.323	2	188	0.0022	0.063	0.228	0.203
10	Hct T1	7.651	2	188	0.0006	0.075	0.275	0.252
11	Hct T6	4.039	2	186	0.0192	0.042	0.215	0.190
12	Hct T18	9.065	2	187	0.0002	0.088	0.295	0.272
13	Fibrinogen T1	6.780	2	187	0.0014	0.068	0.129	0.101
14	Fibrinogen T18	5.904	2	186	0.0033	0.060	0.095	0.066
15	D-dimer T6	2.744	2	185	0.0670	0.029	0.069	0.038
16	CTD volume 1^st^ hour	5.873	2	164	0.0034	0.067	0.102	0.069
17	CTD volume 2^nd^ hour	5.430	2	184	0.0051	0.056	0.087	0.058
18	CTD volume 3^rd^ hour	2.628	2	187	0.0749	0.027	0.053	0.022
19	CTD volume 4^th^ hour	4.908	2	186	0.0084	0.050	0.086	0.057
20	CTD volume 5^th^ hour	8.928	2	184	0.0002	0.088	0.137	0.108
21	CTD volume 6^th^ hour	5.802	2	186	0.036	0.059	0.120	0.092
22	Hb mass CTD 6^th^ hour	5.426	2	185	0.0051	0.55	0.137	0.109
23	Total Hb mass loss via CTD	4.663	2	185	0.0106	0.048	0.141	0.113
24	Intra-op Hb mass loss	79.552	2	188	0.0000	0.458	0.567	0.553
25	Hb mass loss via CTD T1	3.001	2	180	0.0522	0.032	0.068	0.037
26	Hb mass loss via CTD T6	2.914	2	188	0.0567	0.030	0.130	0.102
27	Hct CTD T6	2.795	2	187	0.0636	0.029	0.107	0.079
28	ACT before surgery	2.897	2	186	0.0577	0.030	0.059	0.029
29	Summary CTD volume	2.361	2	186	0.0971	0.025	0.116	0.088

Models include *Gender* and *Age* as covariates; outliers >3 × IQR were removed within each *Blood loss category × Gender* cell. All reported F-tests refer to the main effect of *Blood loss category* (Type III SS). Significance threshold = *p* < 0.05. *Note: Partial eta square η*^*2*^ *(Cohen’s guidelines)*.

• .01 → small

• .06 → medium

• .14 → large

*Note*: ANCOVA – Analysis of Covariance; Hb – haemoglobin; BMI – body mass index; RBC – red blood cells; Hct – haematocrit; CTD – chest tube drainage; ACT – activated clotting time; T0 – preoperative; T1 - immediately postoperative; T6 – 6 h postoperative; T18 – 18 h postoperative; IQR – interquartile range.

### Discriminative performance

The discriminative ability of the derived clusters was assessed using ROC analysis (Figure 1). The model demonstrated varying performance across clusters. The strongest separation was observed in the Severe group (Cluster 3) (AUC = 0.790; 95% CI 0.721–0.859), indicating high efficacy in identifying patients with major Hb/kg index. The Mild group (Cluster 1) demonstrated moderate discriminatory ability (AUC = 0.659; 95% CI 0.579–0.739). The Moderate group (Cluster 2) showed weak separability (AUC = 0.576; 95% CI 0.479–0.673). Overall, the micro-averaged AUC for the model was 0.687, confirming that the selected features effectively stratify the extremes of the blood loss spectrum.

**Figure 1.**
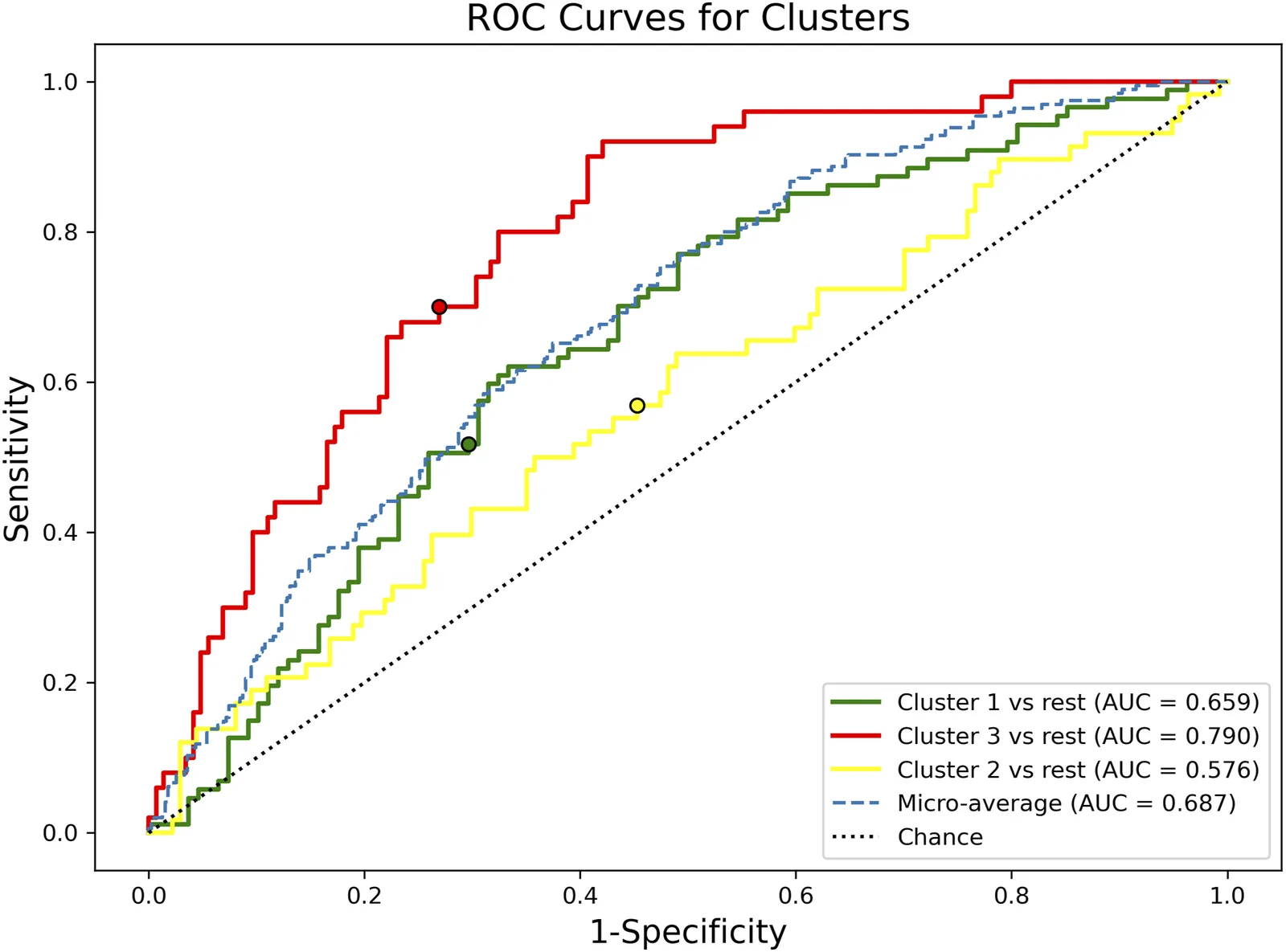
ROC curves for discrimination of blood loss severity clusters.

### Primary outcome: blood loss severity in terms of the Hb/kg Index

Adjusted two-way ANCOVA confirmed a significant difference between blood loss severity clusters (Table 3). The Hb/kg Index demonstrated the strongest association (*F*(2,186) = 250, *p* < 0.001, $\eta_{p}^{2}$ = 0.729) (Figure 2), increasing across clusters (Mild: 1.22 ± 0.38; Moderate: 1.94 ± 0.10; Severe: 2.65 ± 0.36). The model explained 80.3% of the variance (*R*^*2*^ = 0.803). Gender (*p* = 0.757) and the Cluster × Gender interaction (*p* = 0.992) were not significant. Intraoperative Hb mass loss also differed significantly between clusters (*F*(2,188) = 79.55, *p* < 0.001, $\eta_{p}^{2}$ = 0.458), explaining a substantial proportion of the variance (*R*^*2*^ = 0.567; adjusted *R*^*2*^ = 0.553) (Supplemental Figure 1). Mean values were 99 ± 33 g in the Mild, 149 ± 32 g in the Moderate and 187.5 ± 40 g in the Severe groups. Neither gender (*F*(1,188) = 1.63, *p* = 0.203), the Cluster × Gender interaction (*F*(2,188) = 1.11, *p* = 0.333), nor age (*F*(1,188) = 0.33, *p* = 0.569) were statistically significant. Consequently, RBC count at T1 decreased as cluster severity increased (*F*(2,188) = 11.85, *p* < 0.001, $\eta_{p}^{2}$ = 0.112): Mild: 3.81 ± 0.52, Moderate: 3.68 ± 0.43, and Severe: 3.43 ± 0.45 (10^12^/L) (Supplemental Figure 2). The Cluster × Gender interaction was not significant. The model explained 27% of the variance (*R*^*2*^ = 0.250). BMI decreased from the Mild to Severe groups (30.47 ± 5.3, 28.73 ± 4.9, and 26.32 ± 4.1 kg/m^2^; *F*(2,188) = 10.322, *p* < 0.001, $\eta_{p}^{2}$ = 0.099) (Figure 3). Weight varied significantly between groups (*F*(2,188) = 9.81, *p* < 0.001, $\eta_{p}^{2}$ = 0.095) and by gender (*F*(1,188) = 18.45, *p* < 0.001): men 87.9 ± 15.3 kg, women 76.4 ± 16.2 kg (Supplemental Figure 3). Intraoperative parameters, including CPB duration, cross-clamp and reperfusion times, as well as ICU days and hospital stay, showed no significant effects (all *p* > 0.05). Chest Tube Drainage: Volume vs. Content. CTD volume did not differ significantly (Mild 343 ± 175 ml; Moderate 341 ± 171 ml; Severe 427 ± 186 ml; *p* = 0.097, $\eta_{p}^{2}$ = 0.025) (Table 3). In contrast, Hb mass loss via CTD demonstrated statistically significant difference between groups (*p* = 0.011, $\eta_{p}^{2}$ = 0.048): Mild 15 ± 9 g, Moderate 15 ± 10 g and Severe 21 ± 17 g. Furthermore, the *content* of the drainage varied: a significant omnibus effect was found for Hb (χ^2^(2) = 13.468, *p* = 0.001) in the fluid. Associations between the Hb/kg Index and perioperative variables are presented in Supplemental Table 1 and Supplemental Figure 4. Stratified Pearson correlation coefficients are shown in Supplemental Table 2.

**Figure 2.**
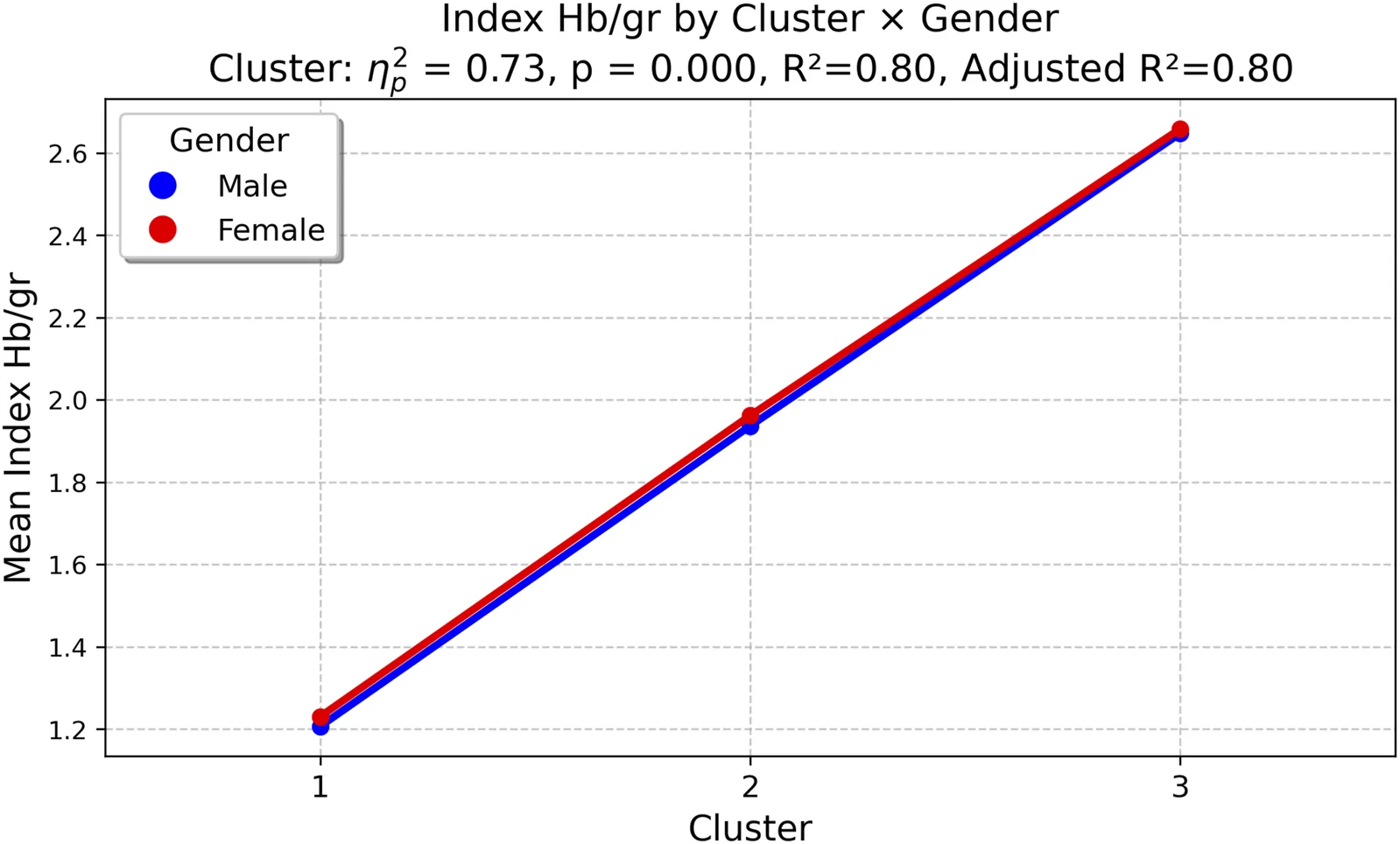
Mean Hb/kg Index across blood loss severity clusters.

**Figure 3.**
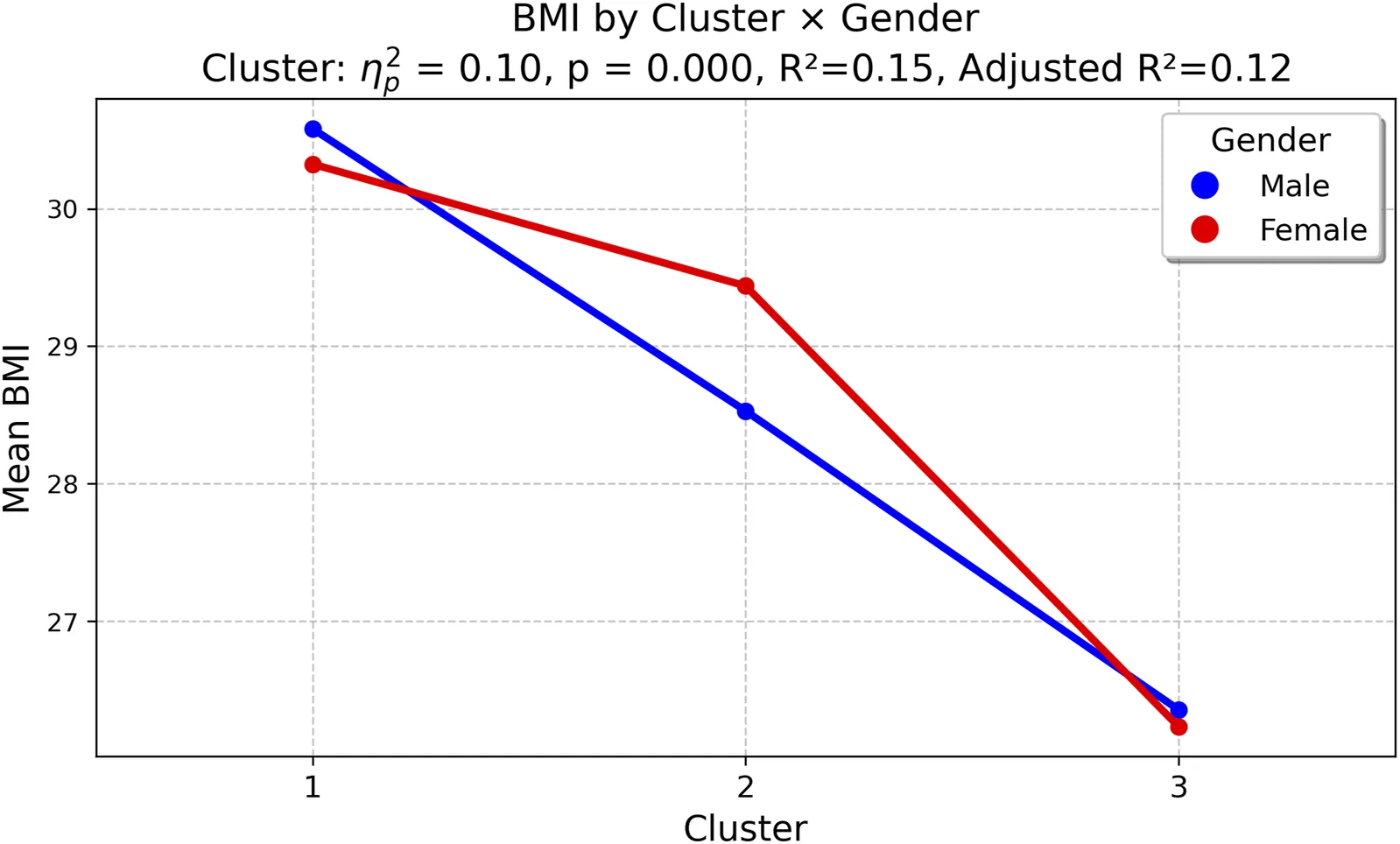
Mean body mass index (BMI) across blood loss severity clusters.

### Gender-specific analysis

A gender-specific analysis compared correlations in men (*n* = 134) and women (*n* = 61). While most variables showed similar patterns, haemoglobin after surgery demonstrated a significantly stronger negative correlation with the Index in women at both T1 (r = −0.53 vs −0.26; *p* = 0.044) and T18 (r = −0.51 vs −0.23; *p* = 0.040). Additionally, ADP platelet aggregation at T18 correlated negatively in men (r = −0.17) but positively in women (r = 0.17) (*p* = 0.033). Interaction analysis identified a notably high rate of heart failure in women within the Moderate cluster (40%; *p* = 0.010).

### Clinical outcomes

The analysis of postoperative complications showed similar morbidity rates across the clusters. The incidence of HF did not differ significantly between groups (*p* = 0.592), nor did the rates of AF (*p* = 0.973) or AKI (*p* = 0.533). Similarly, no significant differences were observed for neurological complications or atrioventricular block (*p* > 0.05).

## Discussion

In this study, we developed and validated a three data-driven blood loss severity clusters based on the Hb/kg Index. The robustness of this clustering was demonstrated by the very large effect size for the Hb/kg Index ( $\eta_{p}^{2}$ = 0.729). This confirms that the clusters are not arbitrary statistical groupings but physiologically meaningful categories reflecting distinct magnitudes of true haemoglobin mass loss. While mild postoperative bleeding is clinically insignificant, severe bleeding after cardiac surgery is associated with catastrophic outcomes,^4,5,19^ prolonged intensive care and increased use of all types of resources.^3,7^ Clinicians face a major challenge in treating patients, as there is no generally accepted method for blood loss measurement,^20^ because there is no universal bleeding classification.^21^ Various definitions of bleeding were proposed to stratify blood loss severity. Most of them are based on visual estimation of CTD volume.^10,11,13,22,23^ However they have significant limitations.^11,24^ Early re-exploration for high chest tube output may limit total blood loss, while late chest tube output may reflect an accumulation of both serum and blood.^13^ Thus, estimation of CTD often under- or overestimates actual blood loss.^18,25,26^ Our findings support this critique. We observed only a weak positive association between the clusters and CTD volume (F = 2.361, *p* = 0.097; $\eta_{p}^{2}$ = 0.025). In contrast, Hb mass loss via CTD showed a significant cluster effect (F = 4.663, *p* = 0.011; ηp^2^ = 0.048), with an effect size nearly twice that of CTD volume. These findings strongly confirm that haemoglobin content in the drained fluid provides a more accurate physiological measure of blood loss than volume alone. A key finding of our stratified analysis is the divergence in the reliability of drainage content. In the Mild cluster, we found a significant positive correlation between the Hb/kg Index and haemoglobin mass in the drainage (r = 0.39), indicating that in standard cases, chest tube output reasonably reflects physiological loss. However, this correlation was absent in the Moderate and Severe clusters. In cases of severe bleeding, haemodilution obscures the true extent of haemorrhage. As a result, the drainage fluid becomes diluted or sequestered, failing to represent the actual intravascular deficit. This paradox demonstrates that relying on drainage composition is misleading in high-risk patients, whereas the Hb/kg Index remains a stable indicator of total mass loss.

The Bleeding Academic Research Consortium (BARC) definition is a binary, high-threshold endpoint that is useful for trials but does not capture the full spectrum of bleeding.^15^ The European Multicenter Study on Coronary Artery Bypass Grafting (E-CABG) and Universal definition of Perioperative Bleeding (UDPB) classification systems are more holistic but are confounded by their reliance on clinical interventions such as transfusions and re-exploration.^13^,^23^ This reliance makes a purely physiological interpretation of bleeding difficult, as intervention thresholds vary. Finally, the Advanced Trauma Life Support (ATLS) classification system categorises haemorrhagic shock in trauma into four classes (I–IV). This model, based on a healthy 70 kg individual, relates the percentage of blood volume loss (from less than 15% to more than 40%) to changes in physiological parameters, including heart rate and blood pressure.^27–29^ However, a common limitation of these systems is the lack of normalization of Hb mass loss per patient weight. The prominent role of weight and BMI in distinguishing bleeding severity underscores a fundamental physiological principle: identical absolute blood volume loss affects patients with different weight and BMI differently. In our study, weight and BMI demonstrated strong and clinically meaningful differences across the clusters. Both weight (F = 9.811, *p* < 0.001, $\eta_{p}^{2}$ = 0.095) and BMI (F = 10.322, *p* < 0.001, $\eta_{p}^{2}$ = 0.099) had medium effect sizes, confirming that patients with lower BMI were markedly overrepresented in the Severe cluster. This pattern is fully consistent with the mathematical structure of the Hb/kg Index and with its biological interpretation: for the same absolute haemoglobin loss, patients with lower body mass experience a higher relative deficit per kilogram and demonstrate disproportionately greater physiological impact. Spearman correlations further supported this relationship, showing significant negative associations between the Hb/kg Index and both weight (ρ = −0.27, *p* < 0.0001) and BMI (ρ = −0.31, *p* < 0.0001). Thus, weight and BMI are not peripheral variables but critical determinants of bleeding severity classification, reaffirming the need for patient-normalised indices rather than volume-based metrics.

Unlike previous studies that established an association between massive bleeding and adverse morbidity,^1,30,31^ our cohort showed no statistically significant differences in the incidence of complications such as HF, AF, or AKI across blood loss severity clusters. This may be due to the modest sample size (*N* = 195) or effective clinical management that mitigated the impact of blood loss on organ function.

Our analysis also highlights important gender-related differences in the physiological response to blood loss. The Hb/kg Index itself proved robust and independent of gender (ANCOVA main effect p = 0.757), which is a key advantage of this standardisation, suggesting the metric quantifies haemoglobin loss comparably in men and women. Nevertheless, women showed significantly stronger negative correlation between the Index and postoperative Hb at both T1 and T18 (*p* = 0.044 and *p* = 0.040, respectively). This correlation is explained by the patients baseline status: women had lower baseline Hb. Furthermore, with a lower estimated blood volume, the same objective Hb/kg loss has a much greater proportional impact on physiological reserve, making circulating Hb a more sensitive and immediate indicator of blood loss in women. We also identified a significant Cluster × Gender interaction for heart failure after surgery (*p* = 0.010). The 40% rate observed in women within the Moderate cluster highlights the need for gender-adjusted risk stratification.

Finally, we observed a fundamentally divergent haemostatic response in platelet function. Although women entered surgery with higher platelet counts (*p* = 0.021), their haemostatic response to blood loss differed fundamentally from that of men. In males, higher Hb/kg values were associated with reduced platelet aggregation (r = −0.17), whereas in females the relationship was positive (r = 0.17), indicating enhanced aggregation with greater blood loss. This significant divergence (*p* = 0.033) suggests a paradoxical, hypercompensatory platelet response in women.

### Conclusion

We validated the Hb/kg Index as a superior, objective metric for blood loss stratification. Unlike conventional volume-based methods, it highlights the vulnerability of patients with lower BMI, often missed by absolute volume metrics, and provides a precise tool for quantifying blood loss.

## Supplemental material

Supplemental Material - Perioperative blood loss in cardiac surgery: Validation of machine learning-derived clusters Supplemental Material for Perioperative blood loss in cardiac surgery: Validation of machine learning-derived clusters by Yerlan Orazymbetov, Serik Aitaliyev, Povilas Jakuška, Audronė Veikutienė, Tadas Lenkutis, Rassul Zhumagaliyev, Aušra Saudargienė and Rimantas Benetis in Perfusion
