# Supplementary material for: Perioperative blood loss in cardiac surgery: Validation of machine learning-derived clusters
Source: Perfusion. 2026 Mar 26;41(8):1420–8. doi: 10.1177/02676591261440347 (PMC13736593; doi:10.1177/02676591261440347)

Detailed statistical analysis. All statistical analyses were performed using IBM SPSS Statistics (version 30.0, IBM Corp., Armonk, NY, USA) and Python 3.10 statistical libraries. Continuous variables were tested for normality using the Shapiro–Wilk test and for homogeneity of variances with Levene's test. Descriptive statistics are reported as mean ± SD for continuous variables and as frequencies (%) for categorical data. Extreme outliers were removed using the 3 × IQR rule within each Cluster × Gender cell to minimise heteroscedasticity and leverage effects before ANCOVA.

Feature selection. Variables for clustering were selected based on their correlation with Hb/kg Index. Spearman's rank correlation was used to evaluate these associations. Only variables demonstrating a statistically significant correlation ($p < 0.05$) were retained for cluster formation. This procedure reduced noise and ensured that only clinically relevant parameters contributed to the clusterization.

Cluster formation and cut-off determination. Patients were grouped into three clusters using the **K-Medoids algorithm,** which partitions data based on pairwise dissimilarities in multidimensional feature space. All variables were **standardized (*z*-score)** before analysis to ensure equal weighting. Each observation was assigned to the cluster defined by the nearest medoid. The resulting clusters were used to derive **data-driven cut-off thresholds** for the Hb/kg Index, calculated as the midpoints between the median values of adjacent clusters. These thresholds represent transitions between increasing levels of blood loss severity:

- Cluster 1: lowest Hb/kg values (Mild blood loss)
- Cluster 2: intermediate Hb/kg values (Moderate blood loss)
- Cluster 3: highest Hb/kg values (Severe blood loss).

ROC analysis was performed to assess the discriminative ability of the Hb/kg index. A multinomial logistic regression model, implemented within a one-vs-rest framework, was fitted using the blood parameters to classify observations into the three clusters. For each cluster, the AUC was estimated,

quantifying the model's ability to distinguish the target cluster from the remaining categories. Class-specific ROC curves were generated to illustrate overall classification performance. Additionally, Chi-square (χ^2) tests were performed to evaluate associations between clusters and categorical variables.

A series of **ANCOVAs** was conducted to evaluate the effects of blood loss category and gender on multiple dependent variables, while controlling for age. Prior to analysis, outliers exceeding three times the interquartile range (3 × IQR) within each Cluster × Gender cell were removed. Each model included age as a covariate, with blood loss category and gender as fixed factors. The models tested for the main effects of blood loss category and gender, as well as their interaction (Blood loss category × Gender). Statistical significance was set at $p < 0.05$. The proportion of explained variance was quantified using R^2 and adjusted R^2, and effect sizes were reported using partial eta squared (η_p^2). Finally, the dependent variables were ranked according to the magnitude of the main effect of blood loss category, using the ηp^2 statistic from each model.

Supplemental Table 1. Spearman's Rank Correlation Coefficients between Perioperative Variables and the Hb/kg Index used for Cluster Selection.

No.	Variables	Spearman's *rho*	*p*-value
	Total Hb mass loss during surgery	0.820	<0.0001
	Hb T0	0.370	<0.0001
	RBC T0	0.240	0.0001
	Summary CTD volume	0.250	<0.0001
	CTD volume 6th hour	0.240	0.001
	CTD volume 5th hour	0.230	0.001
	CTD volume 4th hour	0.200	0.005
	CTD volume 3rd hour	0.190	0.008
	CTD volume 2nd hour	0.170	0.021
	Hb mass loss via CTD	0.170	0.018
	Hb mass loss (T6)	0.160	0.028
	aPTT (T18)	0.150	0.040
	Hb T6	-0.150	0.032
	Hct T6	-0.190	0.007
	Fibrinogen T6	-0.190	0.009
	RBC T6	-0.230	0.001
	Fibrinogen T18	-0.280	<0.001
	Hb T18	-0.280	<0.001

	Hb T1	-0.290	<0.001
	Hb transfusion (PRBC)	-0.290	<0.001
	Weight	-0.270	<0.001
	Hct T1	-0.270	<0.001
	BMI	-0.310	<0.001
	Fibrinogen T1	-0.310	<0.001
	RBC T1	-0.320	<0.001
	RBC T18	-0.340	<0.001
	Hct T18	-0.340	<0.001

Abbreviations: Hb – haemoglobin; RBC – red blood cells; CTD – chest tube drainage; aPTT – activated partial thromboplastin time; Hct – haematocrit; PRBC – packed red blood cells; BMI – body mass index; T0 -preoperative; T1 – immediately postoperative; T6 – 6 hours after surgery; T18 – 18 hours after surgery.

Supplemental Table 2. Pearson Correlation Coefficients between the Hb/kg Index and Perioperative Variables

No.	Variables	Pearson *r*	*p-value*
1	Hb T0	0.45	<0.0001
2	Hb T1	-0.25	0.0004
3	Hb T6	-0.12	0.0944
4	Hb T18	-0.24	0.0006
5	Hct T1	-0.23	0.0013
6	Hct T6	-0.16	0.0239
7	Hct T18	-0.32	<0.0001
8	RBC T0	0.30	<0.0001
9	RBC T1	-0.29	<0.0001
10	RBC T6	-0.19	0.0064
11	RBC T18	-0.32	<0.0001
12	Platelets T1	-0.10	0.1752
13	Platelets T6	-0.01	0.8437
14	Platelets T18	-0.09	0.2357
15	Fibrinogen T1	-0.25	0.0005
16	Fibrinogen T6	-0.14	0.0580
17	Fibrinogen T18	-0.25	0.0006
18	D-dimer T1	-0.06	0.4255
19	D-dimer T6	-0.13	0.0740
20	D-dimer T8	-0.12	0.1070
21	aPTT T1	0.06	0.4244
22	aPTT T6	0.05	0.4787
23	aPTT T18	0.13	0.0638
24	PT T1	0.11	0.1160
25	PT T1	0.05	0.5012
26	PT T1	0.07	0.3053
27	INR T1	0.11	0.1126
28	INR T6	0.04	0.6268
29	INR T18	0.08	0.2781
30	Platelet aggregation (ADP) T1	-0.08	0.2377
31	Platelet aggregation (ADP) T18	-0.05	0.4999

32	Platelet aggregation (Adr) T1	-0.04	0.5414
33	Platelet aggregation (Adr) T18	0.07	0.3173
34	CTD volume 1st hour	0.16	0.0273
35	CTD volume 2nd hour	0.18	0.0121
36	CTD volume 3rd hour	0.15	0.0427
37	CTD volume 4th hour	0.18	0.0098
38	CTD volume 5th hour	0.23	0.0016
39	CTD volume 6th hour	0.22	0.0018
40	Summary CTD volume	0.19	0.0085
41	Hb CTD T1	-0.11	0.1163
42	Hb CTD T6	-0.04	0.6039
43	Hb CTD T18	0.05	0.4862
44	Hct CTD T1	-0.08	0.2430
45	Hct CTD T6	-0.01	0.9116
46	Hct CTD T18	0.00	0.9588

Note: Hb – haemoglobin; Hct – haematocrit; RBC – red blood cells; aPTT – activated partial thromboplastin time; PT – prothrombin time; INR – international normalised ratio; ADP – adenosine diphosphate; Adr – adrenaline; CTD – chest tube drainage;

Supplemental Table 3. Stratified Pearson correlation coefficients for the Hb/kg index across blood loss severity clusters.

Variable	Mild Cluster (n=76)	Moderate Cluster (n=46)	Severe Cluster (n=73)
Hb T0	$r = 0.53$, $p < 0.001$	$r = 0.05$, NS	$r = 0.23$, $p = 0.034$
Hb T1	$r = 0.24$, $p = 0.02$	$r = -0.09$, NS	$r = -0.19$, $p = 0.009$
Hb T6	$r = 0.28$, $p = 0.02$	$r = -0.03$, NS	$r = -0.14$, $p = 0.009$
Hb T18	NS	NS	NS
Hct T1	$r = 0.26$, $p = 0.01$	$r = -0.11$, NS	$r = -0.21$, $p = 0.0046$
Hct T6	$r = 0.23$, $p = 0.05$	$r = -0.08$, NS	$r = -0.15$, NS
Hct T18	NS	NS	$r = -0.25$, $p = 0.027$
RBC T0	$r = 0.34$, $p < 0.05$	$r = 0$, NS	$r = 0.18$, NS
RBC T1, T6 and T18	NS	NS	NS
aPTT T1, T6 and T18	NS	NS	NS
PT T1, T6 and T18	NS	NS	NS
INR T1, T6 and T18	NS	NS	NS
D-dimer T6	$r = 0.20$, NS	$r = -0.11$, NS	$r = -0.11$, NS
D-dimer T1, and T18	NS	NS	NS
Fibrinogen T1, T6 and T18	NS	NS	NS
Platelets T1, T6 and T18	NS	NS	NS
Platelet aggregation (ADP) T1, T18	NS	NS	NS
Platelet aggregation (Adr) T1, T18	NS	NS	NS

Hb CTD T1	*r* = 0.39, *p* = 0.0012	NS	NS
Hb CTD T6, T18	NS	NS	NS
Hct CTD T1	*r* = 0.30, *p* = 0.0065	NS	NS
Hct CTD T6, T18	NS	NS	NS
CTD volume 1-6 hours	NS	NS	NS
Summary CTD volume	NS	NS	NS

Supplemental Figure 1. Mean total haemoglobin mass loss across blood loss severity clusters, stratified by gender

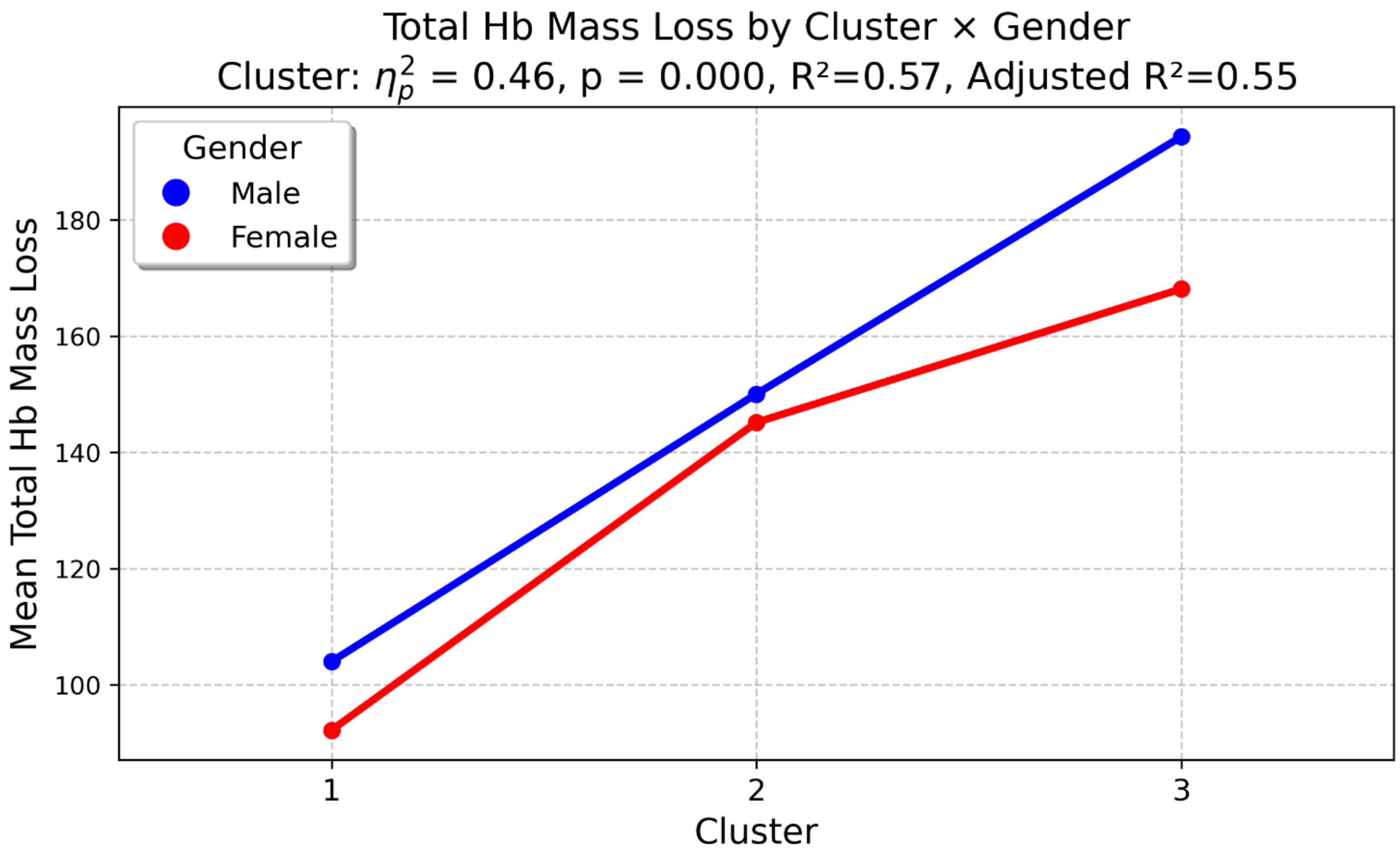

Supplemental Figure 2. Mean red blood cell count at T1 (RBC_T1) across blood loss severity clusters, stratified by gender

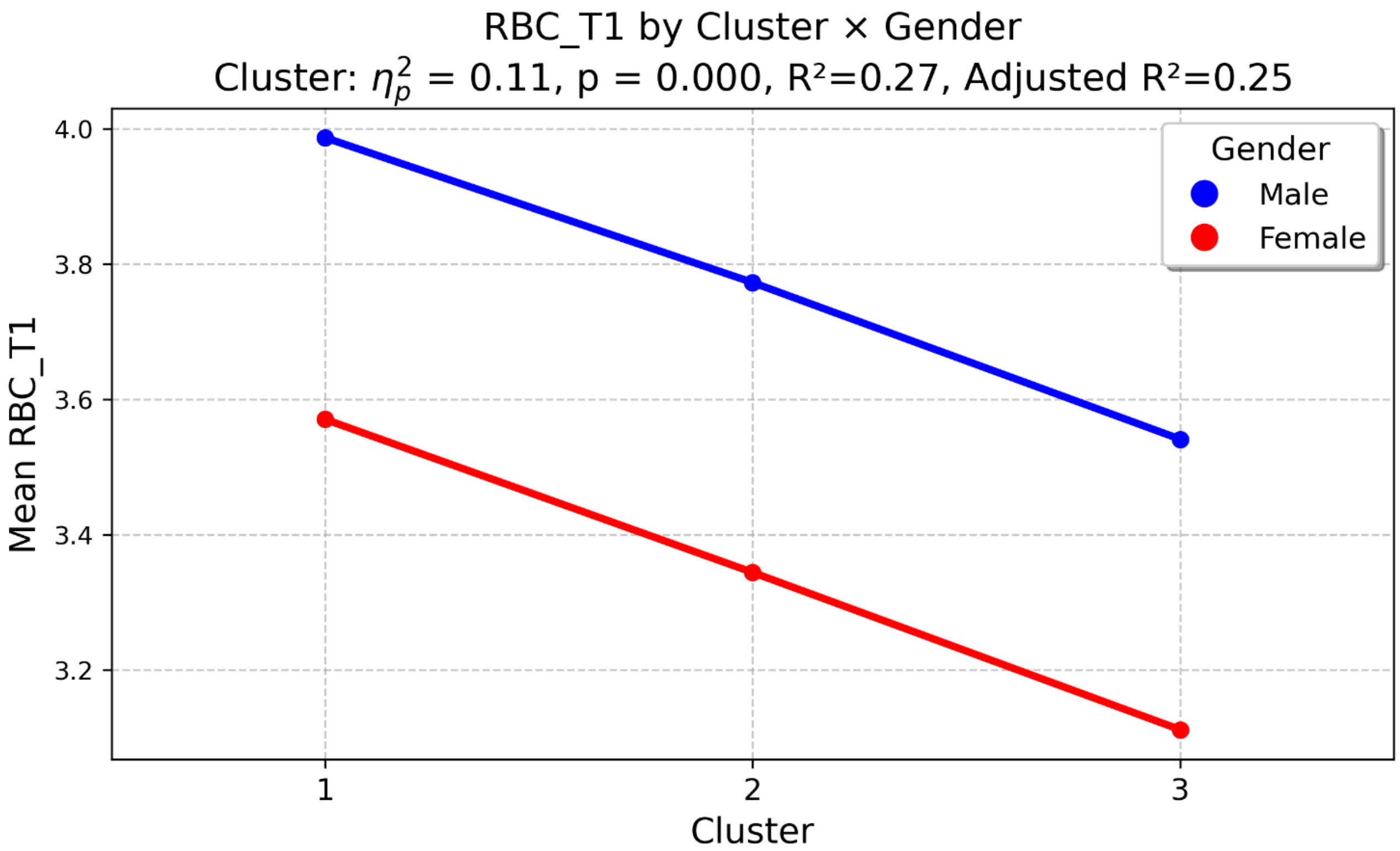

Supplemental Figure 3. Mean body weight across blood loss severity clusters, stratified by sex

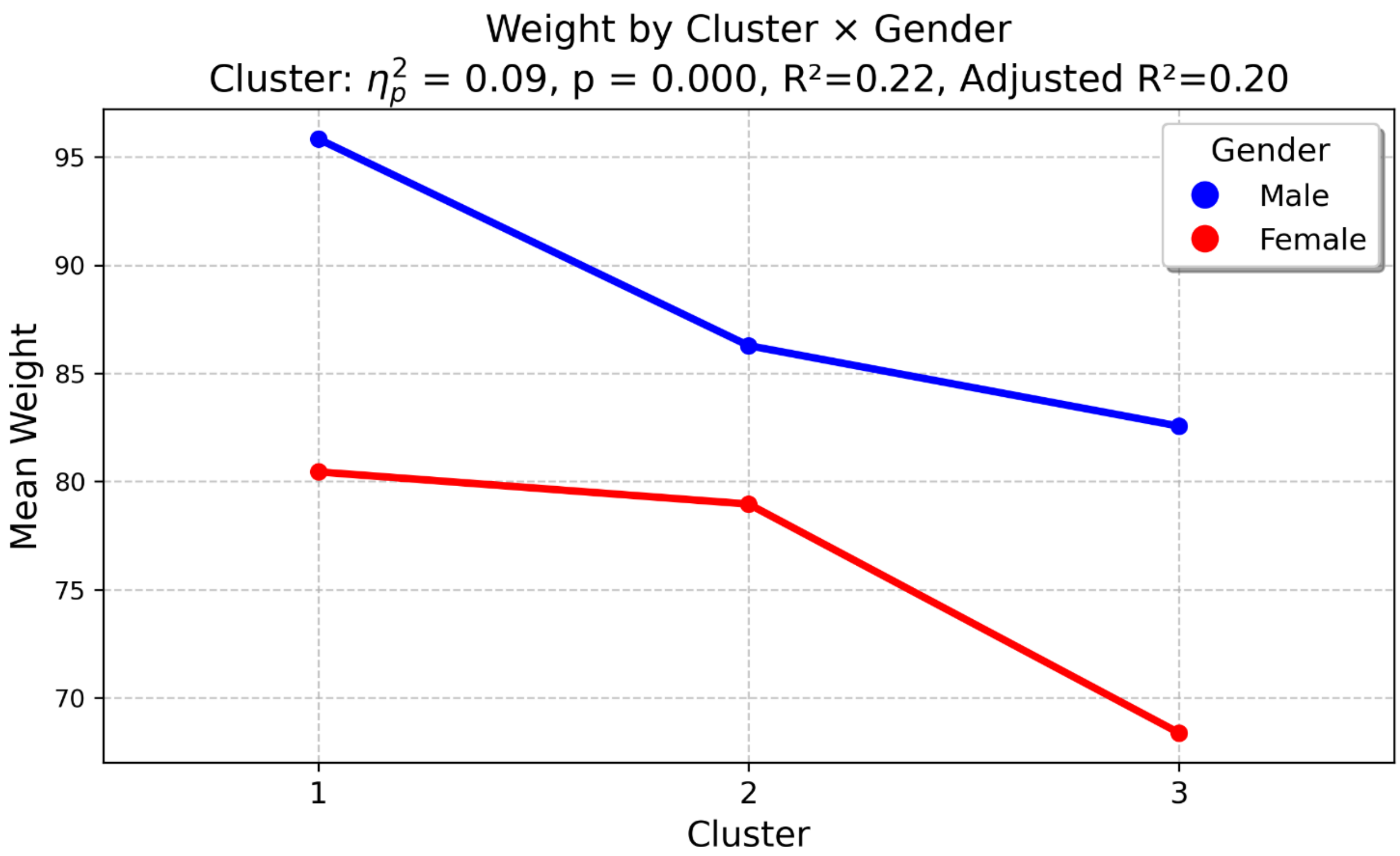

Supplemental Figure 4. Pearson correlation heatmap of the Hb/kg Index and perioperative haematological parameters

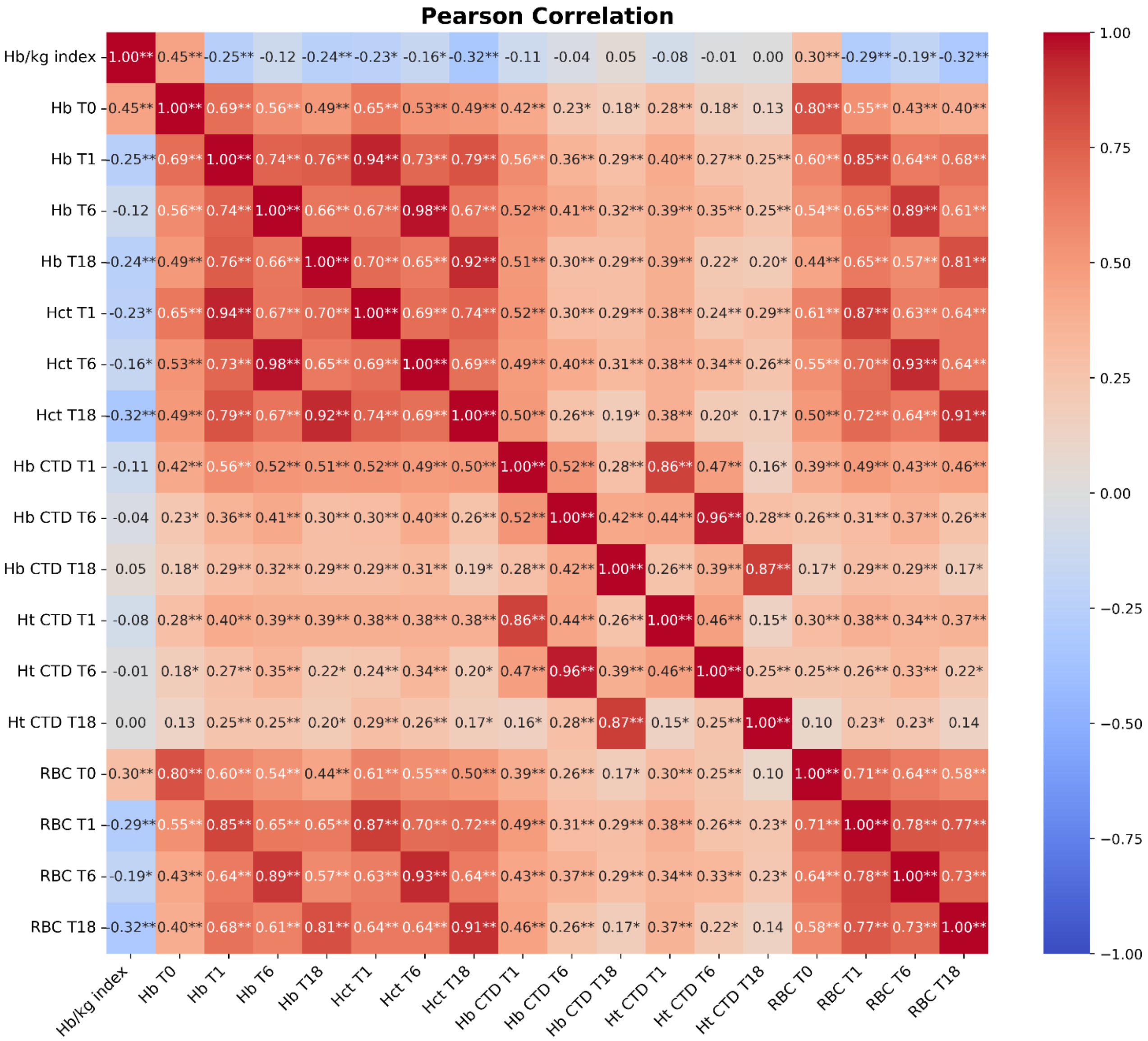

Supplement: Supplementary file 1 [file sj-pdf-1-prf-10.1177_02676591261440347.pdf]
